# MicroRNAs in apoptosis, autophagy and necroptosis

**DOI:** 10.18632/oncotarget.3523

**Published:** 2015-04-02

**Authors:** Zhenyi Su, Zuozhang Yang, Yongqing Xu, Yongbin Chen, Qiang Yu

**Affiliations:** ^1^ Department of Biochemistry and Molecular Biology, Medical School, Southeast University, Nanjing, Jiangsu 210009, China; ^2^ Department of Cell Biology, Harvard Medical School, Boston, MA 02115, USA; ^3^ Bone and Soft Tissue Tumors Research Center of Yunnan Province, Department of Orthopaedics, the Third Affiliated Hospital of Kunming Medical University (Tumor Hospital of Yunnan Province), Kunming, Yunnan 650118, China; ^4^ Department of Orthopaedics, Kunming General Hospital of Chengdu Military Command, Kunming, Yunnan 650118, China; ^5^ Key Laboratory of Animal Models and Human Disease Mechanisms, Kunming Institute of Zoology, Chinese Academy of Sciences, Kunming, Yunnan 650223, China; ^6^ Shanghai Institute of Materia Medica, Chinese Academy of Sciences, Shanghai 201203, China

**Keywords:** microRNA, apoptosis, autophagy, necroptosis, cancer progression

## Abstract

MicroRNAs (miRNAs) are endogenous 22 nt non-coding RNAs that target mRNAs for cleavage or translational repression. Numerous miRNAs regulate programmed cell death including apoptosis, autophagy and necroptosis. We summarize how miRNAs regulate apoptotic, autophagic and necroptotic pathways and cancer progression. We also discuss how miRNAs link different types of cell death.

## INTRODUCTION

Programmed cell death (PCD) is defined as regulated cell death executed by an intracellular program. Apoptosis was traditionally thought to be the only form of programmed cell death. However, in the last decade, programmed cell death has expanded to include autophagy and necroptosis (programmed necrosis). These modes of programmed cell death, especially apoptosis and necroptosis, serve as natural barriers that restrict malignant cells from survival and dissemination.

A breakthrough in cell and molecular biology in the last decade is the discovery of a new class of endogenous, noncoding small RNAs (microRNAs/miRNAs). The function of these miRNAs is to control gene expression by acting on their target mRNAs, leading to either mRNA degradation or translational repression. Numerous miRNAs have been reported to perform specific functions in the regulation of tumor progression and multiple drug resistance [1–8].

In this review, we summarize how miRNAs regulate apoptosis, autophagy, and necroptosis and focus on the impact of these regulatory activities on cancer progression. We also discuss how miRNAs bridge the crosstalk between different types of cell death. At the end of this review, we highlight the future challenges and propose possible research directions in this field based on our current understanding.

### An introduction to microRNAs

MiRNAs are highly conserved, small noncoding RNA molecules that function to regulate a wide variety of cellular processes by interfering with protein expression or mRNA degradation [9]. In mammals, miRNAs are estimated to regulate approximately 50% of all protein-coding genes and play important roles in all types of biological events, including cell proliferation and differentiation, cell fate determination, signal transduction, organ development, host-viral interactions, tumorigenesis and progression [9, 10]. miRNAs act as guides for the miRNA-RNA-induced silencing complex (miRNA-RISC) to specifically regulate their target mRNAs. With few exceptions, the miRNA-binding sites normally lie in the 3′-untranslated regions (3′-UTRs) of mRNAs. The binding of miRNAs to mRNAs involves the Watson-Crick base pairing of miRNA nucleotides 2–8, representing the seed region [11]. The degree of miRNA-mRNA complementarity is considered to be a key factor affecting the regulatory mechanism of miRNA. Perfect complementarity triggers the Ago-catalyzed cleavage of the mRNA strand, whereas central region mismatches exclude this cleavage but inhibit mRNA translation [11, 12].

### MicroRNAs and apoptosis

#### Apoptotic pathways

**Figure 1:**
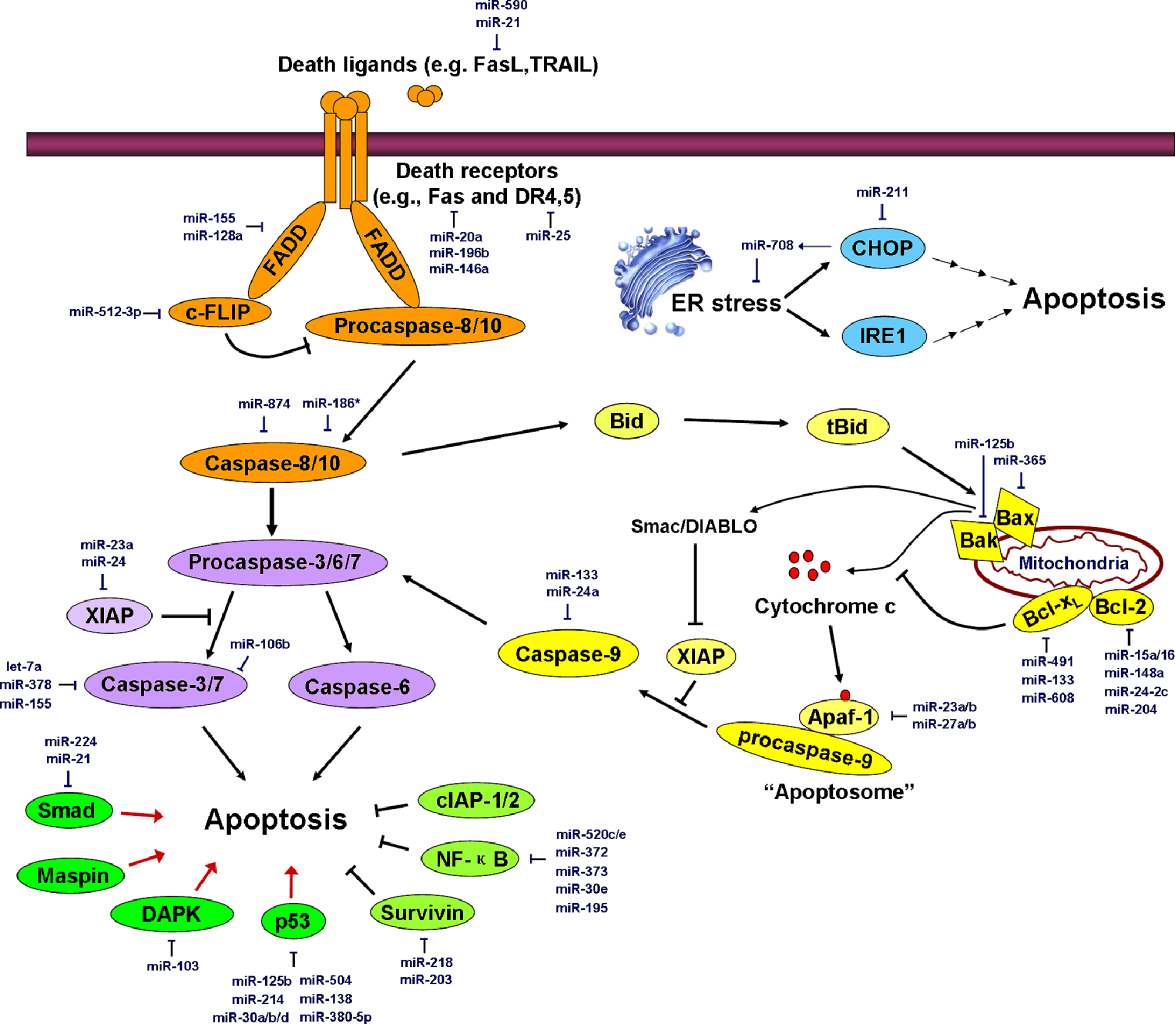
miRNAs regulate the major apoptosis pathways The orange region represents the extrinsic apoptotic pathway; the yellow region represents the intrinsic apoptotic pathway; the blue region represents ER stress-induced apoptosis; the purple region represents common members of different apoptotic pathways; the green region represents critical regulators controlling apoptosis. Major miRNAs that regulate apoptosis effectors are shown in the diagram in dark blue. FasL, Fas ligand; TRAIL, TNF-related apoptosis-inducing ligand; DR4, death receptor 4; FADD, Fas-associated death domain protein; c-FLIP, cellular FLICE-like inhibitory protein; CHOP, C/EBP-homologous protein; IRE1, inositol-requiring protein-1; XIAP, X-linked inhibitor of apoptosis; Maspin, mammary serine protease inhibitor; DAPK, death-associated protein kinase; cIAP1/2, cellular inhibitor of apoptosis 1/2; Smac, second mitochondria-derived activator of caspases, also referred to as DIABLO. See the text for details.

There are two basic apoptotic signaling pathways: the extrinsic and intrinsic apoptotic pathways [13]. The intrinsic (or mitochondrial) apoptotic pathway is triggered by a variety of intracellular stimuli, including DNA damage, cytotoxic drug treatment, growth factor deprivation, and/or oxidative stress. This pathway relies on the formation of a complex termed the apoptosome, which is composed of procaspase-9, apoptotic protease activating factor 1 (Apaf-1), and cytochrome c. A series of Bcl-2 family members including Bax, Bak, Bcl-2, Bcl-x_L_, Mcl-1, Bid, and Bim control the release of cytochrome c by regulating mitochondrial membrane permeabilization. Notably, Bax and Bak homo-oligomerize on the surface of mitochondria to promote pore formation in the outer mitochondrial membrane. Pro-apoptotic factors such as cytochrome c leak out of mitochondria via these pores and play important roles in the induction of apoptosis. The extrinsic pathway of apoptosis is initiated by the binding of death ligands [e.g., Fas ligand (FasL), TNF-related apoptosis-inducing ligand (TRAIL), TNF-α, and TNF-like weak inducer of apoptosis (TWEAK)] to death receptors in the TNF receptor (TNFR) superfamily [e.g., Fas, death receptor (DR)4/5, TNFR, and DR3]. This interaction is followed by the assembly of the death-inducing signaling complex (DISC), which consists of the Fas-associated death domain-containing protein (FADD) and procaspase-8/10. DISC then either activates downstream effector caspases (caspase-3, 6 and 7) to directly induce cell demise or cleaves the Bcl-2 family member Bid to form tBid, thereby triggering the mitochondria-mediated intrinsic apoptotic pathway [13]. In addition to these two well-known apoptotic pathways, chronic endoplasmic reticulum (ER) stress also triggers apoptosis via the activity of inositol-requiring protein-1 (IRE1) and C/EBP-homologous protein (CHOP). These alternative signaling cascades may interact with the intrinsic or extrinsic apoptotic pathway at some points [14]. The major apoptotic processes are depicted in Figure 1.

Numerous factors such as Smads, mammary serine protease inhibitor (Maspin), death-associated protein kinase (DAPK), p53, cellular inhibitor of apoptosis proteins (cIAPs), and NF-κB have been reported to be involved in the regulation of apoptotic pathways [15]. Interestingly, these factors often act at more than one node in either the intrinsic or extrinsic apoptotic pathway. p53, a classic pleiotropic regulator of cell fate in response to DNA damage, trans-activates several pro-apoptotic Bcl-2 family members (Bax, Bid, PUMA, and NOXA), Apaf-1, caspase-6 and -8, the two central death receptors Fas and DR5, and the death ligand TRAIL but trans-represses anti-apoptotic factors such as Bcl-2 and survivin. In addition to transcriptional regulation, p53 directly modulates mitochondrial function by promoting the oligomerization of Bax and Bak and by interacting with Bcl-2, Bcl-x_L_, and Mcl-1 [16, 17].

#### miRNAs that regulate the intrinsic apoptosis pathway

Many miRNAs have been shown to be involved in regulating the intrinsic apoptotic pathway and in inhibiting tumor growth. miR-365, which is highly expressed in invasive ductal adenocarcinoma, directly targets the adaptor protein Src homology 2 domain-containing 1 (SHC1) and the pro-apoptotic protein Bax, and these interactions are associated with gemcitabine resistance in pancreatic cancer cells [18]. miR-125b conferred the resistance of breast cancer cells to paclitaxel via the suppression of pro-apoptotic Bak expression [19]. miR-491 directly targets Bcl-x_L_ and significantly decreases the viability of human DLD-1 colorectal cancer cells by inducing apoptosis. Treatment with miR-491 also suppresses DLD-1 cell-derived tumor growth in nude mice *in vivo* [20]. miR-133a was shown to be down-regulated in osteosarcoma cell lines and in primary human osteosarcoma tissues, and this down-regulation strongly correlated with tumor progression and prognosis. The restoration of miR-133a inhibited cell proliferation, induced apoptosis, and suppressed tumorigenicity in osteosarcoma cell lines. The tumor-suppressive activity of miR-133a is likely due to the targeted suppression of Bcl-x_L_ and Mcl-1 expression [21]. miR-608 was also reported to target Bcl-x_L_ to regulate chordoma malignancy [22]. miR-15a and miR-16–1 were shown to be frequently deleted or down-regulated in a majority of chronic lymphocytic leukemia (CLL) cases, and their expression inversely correlated with Bcl-2 expression. A further study showed that these two miRNAs negatively regulate Bcl-2 at the posttranscriptional level via direct inhibition [23] and induce apoptosis. miR-204 is another suppressor of Bcl-2 expression. The down-regulation of miR-204 correlated with increased Bcl-2 protein staining in human gastric cancer (GC) specimens. The ectopic expression of miR-204 inhibited colony formation, cell migration and tumor engraftment by GC cells and increased the responsiveness of GC cells to 5-fluorouracil or oxaliplatin treatment [24]. miR-148a and miR-24–2c also directly suppress Bcl-2 expression [25, 26]. miR-23a/b and miR-27a/b act as endogenous inhibitors of Apaf-1 expression and control the sensitivity of neurons to apoptosis [27]. miR-133 and miR-24a were observed to directly repress caspase-9 to regulate cell fate (Figure 1) [28, 29].

#### miRNAs that regulate the extrinsic apoptotic pathway

miRNAs are also involved in regulating the extrinsic apoptotic pathway. FasL was reported to be a direct target of miR-21, and the ectopic expression of miR-21 protected cancer cells from gemcitabine-induced apoptosis [30]. miR-590, which is highly expressed in the human acute myeloid leukemia (AML) cell line AML-193, inhibited FasL expression in AML and promoted cell survival [31]. miR-20a down-regulated Fas expression in osteosarcoma cells, thus enhancing the metastatic capacity of osteosarcoma cells by promoting cell survival in the FasL-positive lung microenvironment [32]. miR-146a and miR-196b are also potent suppressors of Fas expression [33, 34]. The ectopic expression of miR-196b led to more aggressive leukemic phenotypes and caused much more rapid leukemogenesis upon secondary transplantation. However, the overexpression of Fas reversed these miR-196b-mediated phenotypes [34].

miR-25 expression is elevated in malignant cholangiocarcinoma cells. It was demonstrated that miR-25 targets the TRAIL receptor DR4 and promotes the evasion of TRAIL-induced apoptosis in cholangiocarcinoma [35]. miR-K10a was reported to prevent TWEAK-induced apoptosis by targeting the TWEAK receptor (TWEAKR) [36]. In contrast to other death ligands, TNF-α can trigger either apoptosis or cell survival depending on its downstream signaling complexes. miR-187, miR-181c, and miR-34a were shown to directly target TNF-α mRNA to regulate apoptosis or inflammation [37–39]. FADD can be transcriptionally regulated by miR-155 [40] or miR-128a [41]. Increased miR-128a levels were frequently observed in acute lymphoblastic leukemia (ALL). The ectopic expression of miR-128a conferred Fas resistance to Jurkat cells by directly targeting FADD, but antagonizing miR-128a function sensitized cells to Fas-mediated apoptosis [41]. Because caspase-8 is a key checkpoint protein that regulates the transition between apoptosis and necroptosis, the suppression of caspase-8 expression by miR-874 promotes the induction of necroptosis [42]. miR-186* was reported to regulate caspase-10, which is highly homologous to casapse-8 [43]. Cellular FLICE-like inhibitory protein long form (c-FLIP_L_), which is highly homologous to procaspase-8 but displays no protease activity, is recruited to DISC and prevents the activation of procaspase-8. Dysregulation of c-FLIP_L_ has been shown to be associated with the malignancy of most human cancers. miR-512–3p negatively regulates c-FLIP expression by acting on a conserved miRNA-binding site in the 3′-UTR of c-FLIP and enhances Taxol-induced apoptosis in HepG2 hepatocellular carcinoma cells (Figure 1) [44].

#### miRNAs that regulate the ER stress-induced apoptotic pathway

miR-211 was shown to be induced by ER stress and to facilitate the histone H3K27 methylation of the CHOP (a key effector that responds to ER-stress) promoter, leading to the suppression of CHOP expression and to cell survival [45]. Interestingly, CHOP up-regulates miR-708, which functions in relieving ER stress and maintaining the homeostasis of ER function (Figure 1) [46].

#### miRNAs that regulate the executioner caspases

Caspase-3, -7, and -6 are effector caspases that act on different apoptotic pathways to execute cell death. miR-let-7a, plays an important role in the modulation of drug-induced apoptosis by targeting caspase-3 in human cancer cells [47]. miR-378 and miR-155 were also reported to target caspase-3 mRNA to attenuate apoptosis [40, 48]. miR-106b, which is up-regulated in both human primary prostate tumors and distant metastases, was shown to target caspase-7 and to be associated with the recurrence of prostate cancer (Figure 1) [49].

#### miRNAs that control the key regulators of apoptosis

DAPK is a classic pro-apoptotic factor and metastasis suppressor. miR-103 and miR-107 promote colorectal cancer metastasis by targeting DAPK and Krüppel-like factor 4 (KLF4) [50]. Smad proteins are critical mediators of TGF-β-induced pro-apoptotic signaling. It was reported that miR-21 binds to the 3′-UTR of Smad7 mRNA to inhibit its translation [51]. miR-224 accelerates hepatoma cell migration and tumor formation by silencing Smad4 [52]. miR-125b was reported to act as a direct negative regulator of p53 and p53-induced apoptosis during development and stress responses [53]. miR-380–5p inhibits p53 expression by binding to a conserved sequence in the p53 3′-UTR. The suppression of endogenous miR-380–5p expression in embryonic stem or neuroblastoma cells increases the p53 level, causing extensive apoptotic cell death. The *in vivo* delivery of a miR-380–5p antagonist diminished the tumor size in a mouse neuroblastoma model [54]. Moreover, miR-214, miR-30a/b/d, miR-504, and miR-138 act as direct negative regulators of human p53 [55–58]. Notably, p53 is also a regulator of several miRNAs, constituting a complex circuit between miRNAs and p53 that is involved in the regulation of various physiological and pathological processes [59–61]. Mutant p53 proteins can also transcriptionally regulate the expression of some miRNAs and can exert oncogenic activities [62].

X-linked inhibitor of apoptosis (XIAP) is a critical apoptosis inhibitor that targets multiple caspase activations. Both miR-23a and miR-24 directly target the 3′-UTR of XIAP mRNA [63]. miR-24 overexpression abolished apoptosis resistance in cancer cells via the suppression of XIAP expression [64]. Survivin, a potent anti-apoptosis regulator, was reported to serve as a direct target of miR-218 and miR-203 [65, 66]. NF-κB is a pivotal protein complex that antagonizes apoptosis and facilitates tumor progression. A series of miRNAs have been demonstrated to be involved in the direct or indirect suppression of NF-κB activity. A few miR-520/373 family members (e.g., miR-372, -373, -520c, and -520e) inhibited NF-κB signaling and abrogated the secretion of the pro-inflammatory cytokines IL-6 and IL-8 by directly targeting RelA [67]. miR-30e* enhanced human glioma cell invasiveness in an orthotopic xenotransplantation model by disrupting the inhibitor of NF-κB alpha (IκBα) [68]. Moreover, miR-195 is involved in the regulation of NF-κB activity by targeting two critical activators of NF-κB, IκB kinase α (IKKα) and TAB3 (Figure 1) [69].

### MicroRNAs and autophagy

#### Autophagy and its regulation

Autophagy is a regulated cellular program that involves the following events: autophagy induction, vesicle nucleation (or formation of a phagophore), vesicle elongation and autophagosome formation, autophagosome retrieval (an intermediate recycling step for the recruitment of lipids and certain regulatory proteins to the growing phagophore), fusion of the autophagosome to a lysosome/endosome and autolysosome formation, and autolysosome cargo degradation [70]. Several molecular complexes or components are very important for the process of autophagosome formation. These components are: (a) the UNC-51-like kinase (ULK) complex; (b) the class III phosphatidylinositol-3 kinase (PI3K) complex; (c) two ubiquitin-like protein conjugation systems, the ATG12 conjugation system and the microtubule-associated protein 1 light chain 3 (LC3) conjugation system; and (d) two transmembrane proteins, autophagy-related gene 9 (ATG9) and vacuole membrane protein 1 (VMP1) [71]. Additionally, p62 is a cargo receptor for ubiquitinated proteins that plays a role in selectively delivering these proteins to the autophagosome [72].

**Figure 2:**
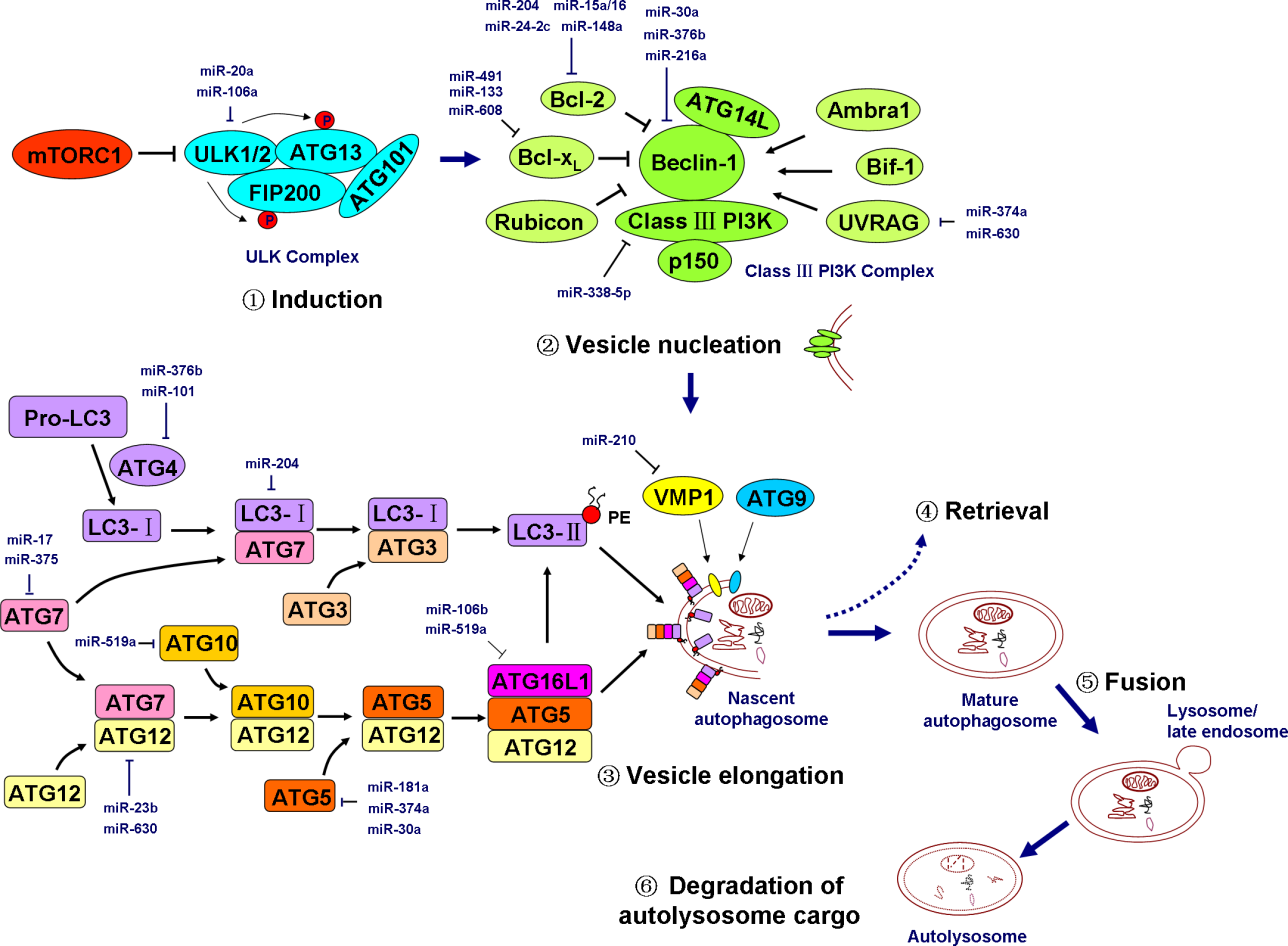
miRNAs regulate the major cascades of autophagy Autophagy includes at least six successive events: induction, vesicle nucleation (or phagophore formation), vesicle elongation and autophagosome formation, ATG protein and lipid retrieval, fusion of the autophagosome to an lysosome/endosome and autolysosome formation, and autolysosome cargo degradation. The ULK complex, which is composed of ULK1/2, ATG13, FIP200, and ATG101, is activated by the inhibition of mTORC1 and initiates the autophagy program. The class III PI3K complex, which is composed of Beclin-1, class III PI3K (i.e., Vps34), p150 (i.e., Vps15), ATG14L, and certain regulatory factors, is essential for vesicle nucleation. Two ubiquitin-like protein conjugation systems form two important complexes (the LC3-II-PE complex and the ATG5-ATG12-ATG16L1 complex) that are critical for vesicle elongation. The transmembrane proteins VMP1 and ATG9 also play a role in nascent autophagosome formation. The major miRNAs involved in the regulation of key members of autophagy cascades are shown in the diagram in dark blue. mTORC1, mammalian target of rapamycin complex 1; ULK, UNC-51-like kinase; ATG, autophagy-related gene; FIP200, focal adhesion kinase family-interacting protein of 200 kDa; UVRAG, UV radiation resistance-associated gene; Rubicon, RUN domain protein as Beclin-1 interacting and cysteine-rich containing; PE, phosphatidylethanolamine; VMP1, vacuole membrane protein 1; PI3K, phosphatidylinositol-3 kinase. Pro-LC3, primary translation product of LC3; LC3-I, cytosolic form of LC3 that is cleaved from Pro-LC3 by ATG4; LC3-II, lipidated form of LC3 that is conjugated to PE. See the text for details.

The ULK complex, which is composed of ULK1, ULK2, ATG13, ATG101, and the focal adhesion kinase family-interacting protein of 200 kDa (FIP200), is crucial for autophagy induction. Mammalian target of rapamycin complex 1 (mTORC1) binds to and inactivates ULK1 and ULK2. The dissociation of mTORC1 from the ULK complex leads to ULK1/2 activation and the subsequent phosphorylation of FIP200 and ATG13, which initiates phagophore formation [73]. The PI3K complex, which is essential for vesicle nucleation, is composed of class III PI3K, Beclin-1, ATG14L, p150, and several regulators including Ambra1, Bif-1, UV radiation resistance-associated gene (UVRAG), Bcl-2, Bcl-x_L_, and the Run domain protein Rubicon [74]. The LC3 and ATG12 ubiquitin-like protein conjugation systems are responsible for vesicle elongation. In the LC3 conjugation system, ATG4 cleaves pro-LC3 into cytosolic LC3-I, which successively binds to ATG7 and ATG3. LC3-I is processed by these two autophagy-related proteins and is then coupled to phosphatidylethanolamine (PE) to become a membrane-bound PE-conjugated LC3, LC3-II. In the ATG12 conjugation system, ATG12 successively binds to ATG7, ATG10, and ATG5 and finally conjugates with autophagy-related 16-like 1 (ATG16L1) to form the ATG16L1-ATG5-AGT12 complex [74, 75]. These two ubiquitination-like systems are closely connected. For example, the ATG16L1-ATG5-AGT12 complex is localized to the phagophore and potentially acts as a novel E3-like enzyme to determine the sites at which LC3 conjugates with PE [76]. VMP1 is thought to function by recruiting Beclin-1 and other components in the class III PI3K complex to the phagophore, whereas ATG9 is thought to contribute to the delivery of membrane particles to the forming autophagosome [74]. The major autophagy cascades are depicted in Figure 2.

Autophagy is a wise strategy to protect cells against potential damage that is triggered by growth signal deficiency, nutrient deprivation (e.g., lack of glucose or amino acids), genotoxic stress (e.g., DNA damage), hypoxic stress, ER stress, and/or ROS accumulation [77].

**Figure 3:**
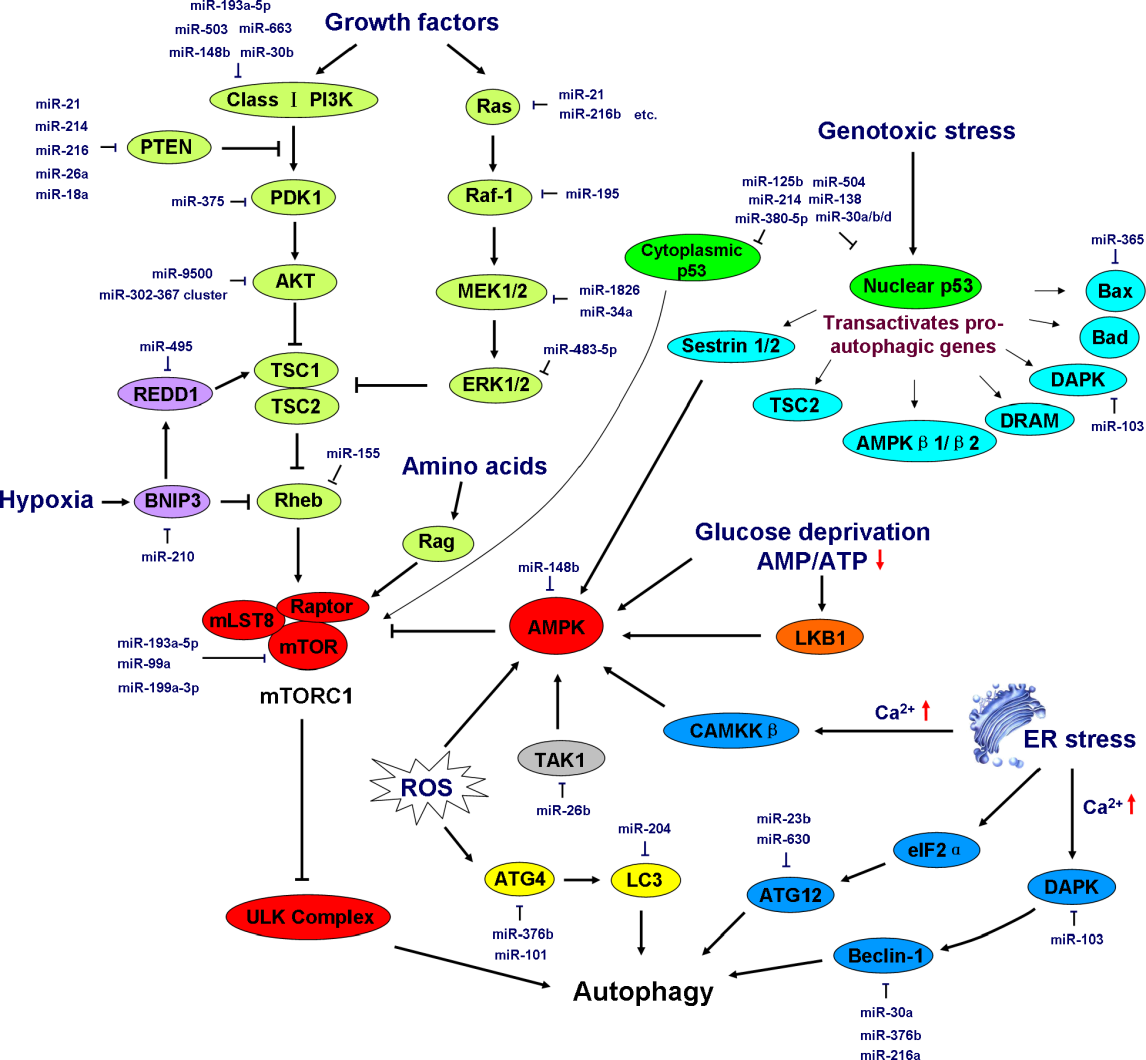
Induction and regulation of autophagy by miRNAs Growth signals, energy status (the abundance of glucose and amino acids), genotoxic stress, hypoxic stress, ER stress, and ROS elicit a series of signaling pathways that initiate or repress autophagy cascades. AMPK-mTORC1 lies at the heart of regulation of autophagy by integrating numerous stimuli and pathways into a signal for the starting point of autophagy, the ULK complex. In addition, ER stress and ROS regulate autophagy independently of the AMPK-mTORC1 pathway. The major miRNAs involved in autophagy regulation are shown in the diagram in dark blue. PDK1, 3-phosphoinositide-dependent protein kinase-1; TSC, tuberous sclerosis complex; Rheb, Ras homolog enriched in brain (a GTPase); REDD1, regulated in development and DNA damage responses 1; BNIP3, Bcl-2/adenovirus E1B 19-kDa-interacting protein 3; mLST8, mammalian lethal with Sec13 protein 8, also referred to as GβL; mTOR, mammalian target of rapamycin; Raptor, regulatory-associated protein of mTOR; MEK, mitogen-activated protein kinase kinase; Rag, a GTPase family member; AMPK, AMP-activated protein kinase; TAK1, TGF-β-activated kinase 1; LKB1, liver kinase B1; DRAM, damage-regulated autophagy modulator; DAPK, death-associated protein kinase; CAMKKβ, calcium/calmodulin kinase kinase β; eIF2α, eukaryotic initiation factor 2α. See the text for details.

The engagement of growth factor receptors activates the class I PI3K complex and a small GTPase, Ras, leading to the activation of the PI3K–PKD1–AKT pathway and the Ras-Raf-1–MEK1/2–ERK1/2 pathways, respectively. Both AKT and ERK1/2 phosphorylate and inhibit tuberous sclerosis complex (TSC) 1/2, thereby stabilizing Ras homolog enriched in brain (Rheb)-GTPase, which, in turn, activates mTORC1, causing the inhibition of autophagy [78]. In addition, high amino acid levels activate Rag GTPase to further activate the regulatory-associated protein of mTOR (Raptor) in mTORC1, leading to the inhibition of autophagy [79]. Glucose deprivation causes an increased AMP:ATP ratio, which activates AMP-activated protein kinase (AMPK) directly or indirectly via liver kinase B1 (LKB1) [75]. Genotoxic stress induces p53 gene expression, and nuclear p53 trans-activates a series of pro-autophagic genes, including sestrin1/2, TSC2, AMPK β1/β2, damage-regulated autophagy modulator (DRAM), DAPK, Bad, and Bax. In contrast to nuclear p53, cytoplasmic p53 plays an opposing role in the induction of autophagy by activating mTORC1 [80]. ER stress also triggers autophagy in a Ca^2+^-dependent or -independent fashion. ER stress-induced Ca^2+^ release activates calcium/calmodulin kinase kinase β (CaMKKβ) and subsequently enhances AMPK activity. Furthermore, Ca^2+^ influx activates DAPK, which phosphorylates and activates Beclin-1, leading to phagophore formation. Moreover, ER stress can trigger autophagy in a Ca^2+^ -independent manner in which ER stress induces eukaryotic initiation factor 2α (eIF2α) phosphorylation and, in turn, up-regulates ATG12 expression [81]. Hypoxic stress triggers autophagy by activating TSC1 or by inhibiting Rheb via Bcl-2/adenovirus E1B 19-kDa-interacting protein 3 (BNIP3) [82]. In addition, ROS acts as a potent inducer of autophagy by activating AMPK or by promoting ATG4 activity to promote LC3 activation [81]. The induction and regulation of autophagy are diagrammed in Figure 3.

#### miRNAs regulate the major autophagy cascades

The ULK complex is situated at the beginning of the autophagy cascades. It was reported that miR-20a and miR-106b participate in the regulation of leucine deprivation-induced autophagy via the suppression of ULK1 expression in myoblasts [83].

Regarding class III PI3K, miR-338–5p was thought to silence the expression of its catalytic subunit (PIK3C3, or Vps34) and suppress autophagy, thereby enhancing colorectal cancer cell migration [84]. Beclin-1, a key component of the class III PI3K complex, is a direct target of miR-30a, miR-376b, and miR-216a. These three miRNAs negatively regulate Beclin-1 expression, causing decreased autophagic activity [85–87]. miR-374a and miR-630 were shown to suppress the expression of UVRAG, a regulator of the class III PI3K complex [88]. The regulatory effects of miRNAs on other effectors (i.e., Bcl-2 and Bcl-x_L_) of the class III PI3K complex have been discussed above.

miRNAs are also involved in the regulation of two conjugation systems. By targeting the LC3 beta isoform (LC3B), miR-204 impeded autophagy and suppressed the growth of renal clear cell carcinoma [89]. ATG4D, an ATG4 family member, is a direct target of miR-101, which was observed to act as a potent inhibitor of basal, etoposide- and rapamycin-induced autophagy [90]. Moreover, miR-376b was shown to silence the expression of the ATG4 family member ATG4C [86]. miR-17 directly interfered with the E1-like enzyme ATG7 and increased the sensitivity of cancer cells to chemotherapy drugs and to low-dose ionizing radiation treatment in human glioblastoma cells [91]. Similarly, miR-375 reduced ATG7 expression by directly targeting and inhibiting autophagy, leading to a reduction in the viability of hepatocellular carcinoma cells under hypoxic conditions [92]. ATG12 is a target of miR-23b. In pancreatic cancer cells, decreased miR-23b expression increased ATG12 expression and autophagic activity, thereby promoting radio-resistance [93]. Additionally, miR-630 was thought to act as another direct suppressor of ATG12 [88]. miR-519a was observed to target the E2-like enzyme ATG10; miR-106b and miR-519a target ATG16L1; and miR-181a, miR-374a, and miR-30a target ATG5 [88, 94, 95].

VMP1, a critical transmembrane protein for phagophore formation, is a direct and functional downstream target of miR-210 that is induced by hypoxia and that augments the metastatic potential of hepatocellular carcinoma cells (Figure 2) [96].

#### miRNAs are involved in the induction and regulation of autophagy

Starvation, genotoxic stress, hypoxic stress, ER stress, and ROS activate a series of signaling pathways to initiate or regulate autophagy cascades. AMPK-mTORC1 serves as the center of autophagy regulation by integrating numerous stimuli and pathways into a signal for the ULK complex, the starting point of autophagy. Several miRNAs have been reported to regulate AMPK-mTORC1. miR-148b was reported to target AMPKα1 to inhibit cell proliferation and invasion and to enhance cancer cell chemosensitivity [97]. miR-26b direct targeted and repressed TGF-β-activated kinase 1 (TAK1, a classic AMPK and NF-κB activator) and other factors to enhance the chemosensitivity of hepatocellular carcinoma cells [98]. Moreover, miR-99a was observed to target mTOR and to suppress the tumorigenicity of lung cancer cells [99]. miR-199a-3p regulated mTOR and c-Met to reduce cell invasion and to increase the sensitivity of human hepatocarcinoma cells to doxorubicin [100].

As for class I PI3K, recent studies showed that the PI3K regulatory subunit p85α (i.e., PIK3R1) serves as a direct target of miR-503. The ectopic expression of miR-503 suppressed non-small-cell lung cancer (NSCLC) cell proliferation and metastasis-related characteristics both *in vitro* and *in vivo* [101]. miR-193a-5p inactivated the AKT/mTOR signaling pathway by directly targeting the class I PI3K regulatory subunit p55γ (i.e., PIK3R3) and mTOR, which suppressed NSCLC metastasis [102]. miR-148b was reported to reduce breast cancer malignancy by coordinating a novel pathway involving the PI3K catalytic subunit p110α (i.e., PIK3CA) [103]. Additionally, the PI3K catalytic subunit p110δ (i.e., PIK3CD) was directly regulated by miR-30b or miR-663 in human colorectal cancer and glioblastoma cells, respectively [104, 105]. Phosphatase and tensin homolog (PTEN), a negative regulator of the PI3K signaling pathway, was demonstrated as a hot target of a number of miRNAs, including miR-21, miR-214, miR-216a, miR-217, miR-26a, and miR-18a, which are involved in the regulation of several cancer types [106–110]. miR-375 was reported to suppress the expression of 3-phosphoinositide-dependent protein kinase-1 (PDK1), another effector in the PI3K signaling pathway that is often down-regulated in gastric carcinoma cases and that is involved in the regulation of cell survival [111]. miR-9500 was shown to regulate the proliferation and migration of human lung cancer cells by targeting AKT1 [112]. In addition, miR-302–367 was observed to be involved in the translational inhibition of AKT1 [113]. Rheb, a negative regulator of autophagy upstream of mTORC1, was silenced by miR-155 via binding to its 3′-UTR, leading to the promotion of autophagy [114, 115]. The major members of the Ras-Raf-1-MEK1/2-ERK1/2 signaling pathway are also under the control of miRNAs. Dozens of miRNAs such as miR-21 and miR-216b [116, 117] were reported to regulate Ras expression and to play a role in tumor suppression^107, 108^. miR-195 was shown to target Raf-1 [118], miR-1826 and miR-34a to MEK1 [119, 120], and miR-483–5p to ERK1 [121].

Hypoxia increases the expression of miR-210, which suppresses BNIP3 expression and maintains cell survival under hypoxic conditions [122]. miR-495 directly suppresses the expression of a downstream component of BNIP3, regulated in development and DNA damage responses 1 (REDD1) and enhances breast cancer stem cell proliferation under hypoxic conditions via a post-transcriptional mechanism [123]. Similarly, other modulators (e.g., ATG4, LC3, DAPK, Beclin-1, ATG12, and Bax) of the ER stress-, ROS-, and genotoxic stress-induced autophagy pathways are regulated by miRNAs; most of these modulators have been described above (Figure 3).

### MicroRNAs and necroptosis

**Figure 4:**
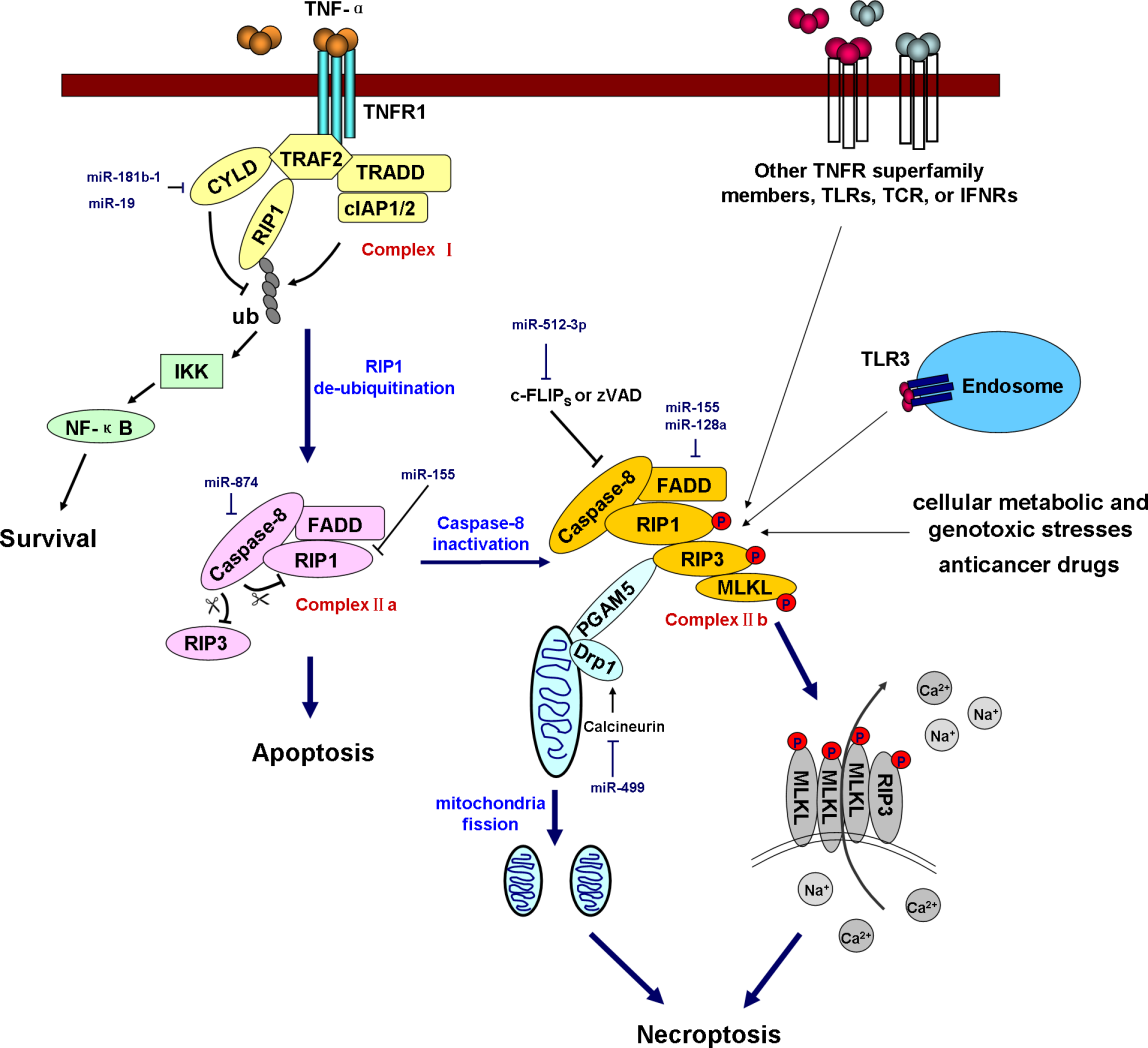
miRNAs regulate necroptosis Necroptosis is triggered by TNF receptor superfamily members, TLRs, IFNRs, TCR, cellular metabolic and genotoxic stresses, and anticancer drugs. In TNF-α-induced necroptosis, the engagement of TNFR1 recruits Complex I (composed of TRADD, RIP1, TRAF2, CYLD, and cIAP1/2). In this complex, cIAP1 and cIAP2 ubiquitinate RIP1, whereas CYLD deubiquitinates RIP1. Polyubiquitinated RIP1 promotes NF-κB activation and prevents the formation of Complex II a (composed of caspase-8, FADD, RIP1) and Complex II b (composed of caspase-8, FADD, RIP1, RIP3, and MLKL), thus promoting cell survival and inhibiting apoptosis and necroptosis. RIP1 deubiquitination enables Complex II a formation, but whether the cell undergoes necroptosis is dependent on caspase-8 activity. Activated caspase-8 cleaves RIP1 and RIP3 and blocks necroptosis. However, if caspase-8 is inactivated by a pharmacological inhibitor (e.g., zVAD), an endogenous inhibitor (e.g., c-FLIP_S_) or genetic caspase-8 or FADD inhibition/deletion, it loses the capacity to cleave RIP1 and RIP3, leading to the trans-phosphorylation of these two kinases and the formation of a filamentous-like complex termed the necrosome. The RIP1-RIP3 necrosome subsequently recruits and activates MLKL and PGAM5. MLKL is phosphorylated by RIP3 and then forms a homotrimer that translocates to the plasma membrane, and this event leads to necrotic plasma membrane permeabilization. Upon necrosis induction, PGAM5S recruits and activates Drp1, which leads to mitochondrial fission, which is an early and necessary step for necrosis execution. The miRNAs involved in the regulation of key components of the necroptotic pathway are shown in the diagram in dark blue. TLRs, Toll-like receptors; TCR, T-cell receptor; IFNRs, interferon receptors; TNFR1, TNF-α receptor 1; TRADD, TNFR1-associated death domain protein; TRAF2, TNF receptor-associated factor 2; CYLD, cylindromatosis; cIAP1/2, cellular inhibitor of apoptosis 1/2; RIP1, receptor-interacting protein kinase 1, also referred to as RIPK1; IKK, IκB kinase; c-FLIP_S_, cellular FLICE-like inhibitory protein, short form. PGAM5, phosphoglycerate mutase 5; MLKL, mixed lineage kinase domain-like; Drp1, dynamin-related protein 1.

Necrosis was traditionally thought to be an accidental and unregulated type of cell death. However, emerging evidence has shown that necrosis can be induced and regulated in a similar manner to apoptosis. Regulated necrosis is termed “programmed necrosis” or “necroptosis” to distinguish this process from necrosis induced by physical trauma [124]. Necroptosis can be induced by the engagement of the TNF receptor superfamily, T-cell receptor (TCR), interferon receptors (IFNRs), Toll-like receptors (TLRs), cellular metabolic and genotoxic stresses, and a number of anti-cancer agents [125]. Necroptosis can be pharmacologically inhibited using certain chemical compounds such as necrostatin-1 [126]. The formation of the “necrosome” by receptor-interacting protein kinase 1 (RIP1, also known as RIPK1) and RIP3 is the most critical event in necroptosis. Multiple stimuli and pathways that induce necroptosis ultimately converge at the RIP1-RIP3 necrosome. In TNF-α-induced necroptosis, the engagement of the TNF receptor recruits a series of proteins including TNFR1-associated death domain protein (TRADD), RIP1, TNF receptor-associated factor 2 (TRAF2), deubiquitinase cylindromatosis (CYLD), and cIAP1/2 to form a large complex termed Complex I. In this complex, cIAP1 and cIAP2 ubiquitinate RIP1 and prevent its entrance into Complex II a (composed of caspase-8, FADD, and RIP1) and Complex II b [composed of caspase-8, FADD, RIP1, RIP3, and mixed lineage kinase domain-like (MLKL)] [127], thus restricting subsequent apoptosis or necroptosis. Moreover, the poly-ubiquitination of RIP1 promotes NF-κB activation and cell survival. In contrast, CYLD is a deubiquitinating enzyme that is responsible for the removal of ubiquitin chains from RIP1 and that promotes the formation of ComplexIIa. Subsequently, caspase-8 cleaves RIP1 and RIP3, thus preventing their trans-phosphorylation and their phosphorylation of downstream necroptotic factors. However, if caspase-8 is inactivated by pharmacological inhibitors (e.g., zVAD), endogenous inhibitors (e.g., c-FLIP_S_), or genetic caspase-8 or FADD inhibition/deletion, it loses its capacity to cleave RIP1 and RIP3, leading to the phosphorylation of these two kinases and the formation of the filamentous-like RIP1-RIP3 complex, or the necrosome. The RIP1-RIP3 necrosome subsequently recruits and activates MLKL and phosphoglycerate mutase 5 (PGAM5), two critical downstream targets of RIP3 [128, 129]. During TNF-induced necroptosis, MLKL is phosphorylated by RIP3, forms a homotrimer via its amino-terminal coiled-coil domain, and translocates to the plasma membrane, and these events lead to necrotic plasma membrane permeabilization [130]. Upon necrosis induction, PGAM5S (a short form of PGAM5) recruits the mitochondrial fission factor dynamin-related protein 1 (Drp1) and activates its GTPase activity by dephosphorylating Drp1 at serine 637. Drp1 activation leads to mitochondrial fission, which is an early and necessary step for necrosis execution (shown in Figure 4) [131].

At present, few studies have reported on how miRNAs regulate necroptotic cell death. miR-155 has been reported to prevent necroptosis in human cardiomyocyte progenitor cells by directly targeting RIP1 [132]. miR-499 can suppress the calcineurin-mediated dephosphorylation of Drp1, thus inhibiting Drp1 accumulation in mitochondria and Drp1-mediated mitochondrial fission [133]. CLYD, the key deubiquitinating enzyme in the apoptosis/necroptosis pathway, was directly targeted by miR-181b-1 and miR-19, resulting in the hyperactivation of the NF-κB signaling pathway and in a high inflammatory status in cancer cells; these events contribute to tumor progression [134, 135]. In addition, miR-874 was reported to enhance necroptosis by targeting caspase-8, which acts as a key modulator of the transition between apoptosis and necroptosis [42]. To date, no report has demonstrated how miRNAs regulate other key necroptotic factors, including RIP3, MLKL, and PGAM5 (Figure 4).

### MicroRNAs regulate the crosstalk between apoptosis, autophagy, and necroptosis

The molecular mechanisms underlying the interaction between apoptosis, autophagy and necroptosis involve several key nodes including Bcl-2, Beclin-1, ATG3, cFLIP, cIAPs, ATG5, Bcl-x_L_, JNK1, p53, p62, caspase-3, caspase-8, and DAPK. For example, under nutrient-rich conditions in which autophagy is unnecessary, Bcl-2 and Bcl-x_L_ bind to and inhibit the autophagy inducer Beclin-1 [136]. However, when cells are subjected to starvation, the binding activity of Bcl-2 and Bcl-x_L_ is weakened by JNK1-mediated Bcl-2 phosphorylation to initiate autophagy, which promotes cell survival [137]. Similarly, the pro-apoptotic factor DAPK phosphorylates Beclin-1, promoting its dissociation from Bcl-2 and activating autophagy [138]. Caspase-3, -7, and -8 cleave two crucial components of the autophagy-inducing complex, Beclin-1 and Class III PI3K. The cleaved fragments of Beclin-1 lose their capacity to induce autophagy. Notably, the C-terminal fragment of Beclin-1 predominantly localizes to mitochondria and sensitizes cells to apoptosis [139]. Similarly, the cleavage of the autophagic protein ATG5 by the pro-apoptotic factor calpain can also facilitate the conversion from autophagy to apoptosis. Truncated ATG5 translocates from the cytosol to mitochondria and associates with the anti-apoptotic molecule Bcl-x_L_, inducing cytochrome c release and caspase activation [140, 141]. p62, a factor that is responsible for packaging misfolded/damaged proteins into the autophagosome, has been shown to be involved in apoptosis induction by targeting proteasomal degradation [142]. The tumor suppressor p53 plays a vital role in the regulation of apoptosis cascades. However, accumulating evidence supports the additional function of p53 in the regulation of autophagy via different pathways depending on its subcellular localization. On one hand, nuclear p53 functions as a transcription factor to trans-activate several pro-autophagic genes (e.g., DRAM) [143]; on the other hand, cytoplasmic p53 tends to repress autophagy [80].

**Figure 5:**
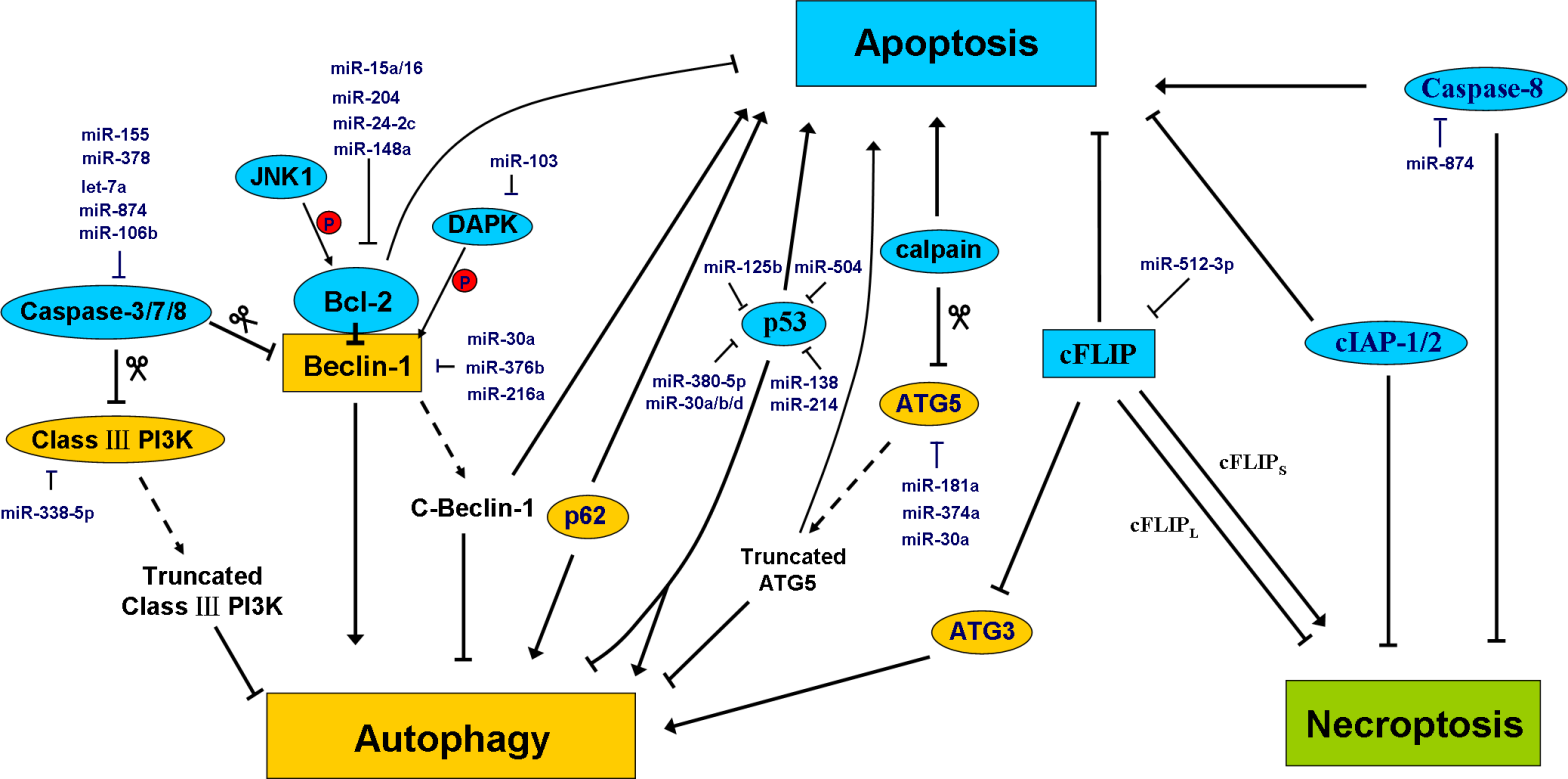
MiRNAs regulate the crosstalk between apoptosis, autophagy, and necroptosis Accumulating studies have shown that a close interaction between apoptosis, autophagy, and necroptosis. Some proteins that are conventionally thought to participate in apoptosis (blue) may play novel roles in autophagy or necroptosis. Alternatively, some autophagy modulators (yellow) may play a role in other modes of programmed cell death. The major miRNAs involved in the regulation of the crosstalk between apoptosis, autophagy, and necroptosis are shown in the diagram in dark blue. See the text for details.

cFLIP, a negative regulator of caspase-8, binds to ATG3 and blocks its conjugation to LC3, thereby attenuating autophagy [144]. In addition, cFLIP mediates the transition between apoptosis and necroptosis. In the absence of cFLIP, cells undergo apoptosis rather than necroptosis due to the high activity of caspase-8. A long isoform of cFLIP (cFLIP_L_) binds to caspase-8 to inhibit caspase-8-mediated apoptosis cascades. However, this cFLIP_L_-caspase-8 heterodimer retains the ability to cleave RIP1, which blocks RIP1–RIP3 formation and necroptosis [145]. In contrast, a short isoform of cFLIP (cFLIP_S_) is able to bind to caspase-8 to inhibit apoptosis, but the cFLIP_S_-caspase-8 heterodimer lacks the capacity to cleave RIP1, leading to the formation of the RIP1–RIP3 complex and the initiation of necroptosis. This reveals a triple role of FLIP, which controls apoptosis, autophagy, and necroptosis at the same time. The E3 ligases cIAP1 and cIAP2 mediate RIP1 ubiquitination, which not only promotes NF-κB activation but also inhibits the binding of RIP1 to Complex II, leading to the suppression of apoptosis and necroptosis [146]. The miRNAs involved in the regulation of this crosstalk are depicted in Figure 5.

### Perspectives

In this review, we have summarized and discussed how miRNAs regulate apoptosis, autophagy, and necroptosis and have listed their roles in cancer progression. However, a few important questions remain to be answered.

(1) To date, limited studies have examined how miRNAs are involved in the regulation of necroptosis. No study has reported on whether and how certain key necroptotic pathway components such as RIP3, MLKL, and PGAM are influenced by miRNAs.

(2) One miRNA may target more than one component of a death pathway and even more than one death pathway. For example, miR-30a targets Beclin-1, ATG5, and p53; miR-155 regulates FADD, caspase-3, Rheb, and RIPK1. It is necessary to understand the crosstalk between these different regulatory activities and the strength of each regulatory effect.

(3) Because targeting cell death pathways represents a promising strategy to treat cancer, the potential clinical applications of miRNAs warrant investigation.
